# Challenges in conducting natural experiments in parks—lessons from the REVAMP study

**DOI:** 10.1186/s12966-016-0460-0

**Published:** 2017-01-17

**Authors:** Jenny Veitch, Jo Salmon, Billie Giles-Corti, David Crawford, Kate Dullaghan, Alison Carver, Anna Timperio

**Affiliations:** 1 Institute for Physical Activity and Nutrition (IPAN), Deakin University, Geelong, Australia; 2 Melbourne School of Population and Global Health, The University of Melbourne, Melbourne, Australia; 3 School of Exercise and Nutrition Sciences, Deakin University, Geelong, Australia

**Keywords:** Natural experiment, Parks, Challenges, Methodology

## Abstract

**Abstract:**

Experimental evidence on the role of the built environment for promoting physical activity is important for informing how to create cities that promote active living. Parks provide opportunities for physical activity; however, there is little robust evidence on the impact of park refurbishment. Government agencies often modify parks, providing opportunities for natural experiment studies of these interventions. Such an opportunity was provided by the modification of a large park in Victoria, Australia in 2013 when the Recording and EValuating Activity in a Modified Park (REVAMP) study was established. Based on insights from the REVAMP study, this paper discusses challenges involved in conducting natural experiments in park settings, focussing on issues that may help design more effective future evaluations of the impact of park refurbishment. Natural experiments offer unique opportunities to evaluate the impact of large-scale changes to the built environment. They provide valuable data that might not otherwise be possible to gather, because of the costs associated with modification of the environment. However, factors beyond the control of the study team contribute to the complexity of both organising and conducting natural experiments, with potential flow-on effects to the quality of data. Therefore many extraneous factors need to be considered when designing, costing and conducting natural experiments; studies should identify opportunities to include key partners from the inception of the project, be flexible yet robust, and allow sufficient funding to accommodate unexpected changes in the research protocol.

**Trial Registration:**

Current controlled trial ISRCTN50745547, registration date 11.1.2014

## Background

The environment in which we live, work and play is widely recognised as having an important influence on our health behaviours [1]. The built environment incorporates features of the neighbourhood environment that are created or modified by people, including homes, schools and workplaces, parks and public open spaces, recreation areas, transport systems, and other design features, such as lighting, street layout and pedestrian crossings [2].

Supportive built environments that facilitate active living have the potential to significantly increase population-levels of physical activity and help prevent many chronic diseases [3]. However, the built environment can also present barriers to physical activity, which make it harder to make active living choices. In an ideal world, researchers would undertake randomised controlled trials (RCTs) to better understand how positive changes to the built environment may reduce barriers and benefit physical activity. For most environmental interventions which are implemented in the real world this ‘gold standard’ study design for demonstrating causality is not feasible as it is not possible to randomly allocate populations to intervention groups. Although relatively new to public health research [4–7], natural experiments evaluating the effectiveness of ‘real world’ changes in the physical environment that are not manipulated by the researcher, provide an alternative study design for investigating causal associations between the built environment and physical activity [8, 9]. Yet, although seen as a priority, natural experiments are infrequently conducted [10].

Public open spaces or parks are important settings within the neighbourhood environment that provide opportunities for physical activity across the lifespan. Given that physical inactivity is a major contributor to the burden of chronic disease [11] attracting residents to parks and encouraging park visitors to be physically active is an important public health goal. Yet, there is little robust intervention study evidence to support park refurbishment or renewal as a means of increasing physical activity [4]. Natural experiments of these interventions provide an appropriate study design for understanding the impact of park refurbishment on park visitation and park-based physical activity.

Conducting natural experiments requires specialist expertise and knowledge and often present conceptual and methodological obstacles [12]. This paper aims to use a natural experiment as a case study to provide an overview of some of the challenges involved in conducting natural experiments. It focusses on issues that may help researchers and planners collaborate on the design of more effective future evaluations of natural experiments, particularly those evaluating the impact of changes to the built environment on population physical activity levels. Although the Medical Research Council has published guidance for conducting natural experiments (www.mrc.ac.uk/naturalexperimentsguidance) [8], this paper provides context specific examples about potential problems and considerations that emerged from conducting a natural experiment in a park. Our observations may or may not apply to natural experiments in other settings.

### Overview of REVAMP: a natural experiment case study

The Recording and EValuating Activity in a Modified Park (REVAMP) study is a natural experiment that examined whether park improvement increased overall park usage, park-based physical activity and active travel to and from an intervention park compared with a control park over 2 years.

The researchers became aware of the proposed refurbishment of the intervention park through a well-established relationship with Parks Victoria. Once the potential to conduct a natural experiment involving this park was established, the researchers invited the participation of three other relevant organisations. Thus, from the outset the study included four partners: the organisation responsible for managing both the intervention and control parks (Parks Victoria); the Victorian Health Promotion Foundation (VicHealth) which is committed to increasing participation in physical activity, creating environments that improve health and reducing health inequalities throughout the community; the water retailer responsible for the region where the intervention park is located (City West Water); and the local council within which the park to be refurbished was located (Brimbank City Council). The inclusion of funding bodies and/or industry as partners with an ongoing involvement in the study confers a sense of study ownership, provides researchers with more insight into the intervention, and facilitates research translation [13]. From the partner’s perspective, being involved in a research project enables them to be at the forefront of research that is well aligned with their organisational priorities; contribute to community consultations, study design, measures, and dissemination strategies; and support, foster and connect with other organisations.

As described elsewhere [14], the intervention park was refurbished from September 2013–February 2014 and data were collected over three time-points in both parks: T1 (baseline, April–May 2013); T2 (April–May 2014); and T3 (April–May 2015) to evaluate its impact. This enabled effects of the refurbishment to be measured over time. The intervention park (329 ha) was located 28 km north-west of Melbourne’s central business district (CBD) in a low socio-economic status (SES) area. In order to determine the impact of the refurbishment, a somewhat comparable control park (120 ha) was identified located 22 km east of Melbourne’s CBD in a high SES area. Both parks included a river and walking/bicycle trails connected with the broader Melbourne trail network. Measures included observations of park visitors in specified target areas from 7.30 am–4.30 pm on four weekdays and 8.30 am–4.30 pm on four weekend days using a modified version of SOPARC (the System for Observing Play and Recreation in Communities) [15], intercept interviews with park visitors, objective measures of usage of walking paths within the park and counts of traffic entering the park at each time-point, and surveys with local residents at T1 and T3.

## Considerations

The conduct of REVAMP provides insights that can be incorporated into the design, costing and management of future natural experiment studies, particularly in park settings but also potentially in other built environment studies such as the construction of new urban greenways and trails [16–18], sporting fields [19], schoolyard playgrounds [20], paths and cycleways [21], and transport infrastructure [22–24]. Specific study experiences and lessons learnt for future studies are described below and summarised in Table 1.

**Table 1:** Summary of study experiences and implications for future studies

Study specific experiences	Lessons learnt/implications for future studies
Partnerships: The inclusion of industry partners had multiple benefits including study ownership, greater insight into the intervention, and facilitation of research translation.	Consider the inclusion of partners prior to the commencement of the project.
Flexibility: Extraneous factors affected the timing and delivery of the intervention in this natural experiment and changes were required to the timeline and study design.	These study designs need to be scientifically robust, yet flexible enough to cope with unpredictable events and a changing environment that is outside the control of the researcher.
Identification of suitable controls: Identification of a control site with similar characteristics to the intervention site (e.g. size, features, area level SES), with no planned changes over the study period was challenging.	Although challenging, an adequate control site is essential to ensure experimental design. It may be necessary to relax the control site criteria rather than have no control site.
Data collection: Factors such as weather, staffing and special events impacted data collection scheduling.	To ensure data collection is unaffected, establish clear study protocols on cancelling observation days, staff schedules, and have contingency plans when these events occur.
Contingency budget: Additional costs were incurred due to changes outside of the researcher’s control (e.g. changes in timelines and rescheduling of data collection due to poor weather).	Incorporate contingency funding into research budgets for natural experiments or alternatively, funders should allow researchers to apply for additional funding to support unanticipated changes outside of their control.
Timing of funding cycles: The timing of the natural experiment was carefully planned to align with university/government funding cycles.	Researchers need to plan in advance to allow for long lead times before the commencement of interventions; however, funders could have flexible funding rounds to accommodate natural experiment evaluations.

***Flexibility is critical:*** *the intervention is not under the researcher’s control*

The inherent nature of a natural experiment means that the researcher has not planned or is not involved in implementing the experimental conditions, but rather has identified an opportunity to evaluate a change in the built, social or policy environment that may have otherwise not been typically evaluated. Researchers are therefore at the mercy of numerous factors outside of their control, such as changes to timelines and/or budgets for implementation of infrastructure [5].

In the REVAMP study, the planned refurbishment in the intervention park was initially scheduled to take place approximately 2 years prior to when it actually commenced. This presented issues for securing funding and recruiting and managing staff. Delays such as this have been observed in other natural experiments. For example, the delay in the building of a new trail had effects on timelines meaning schedules for follow-up measures were affected [18].

Another challenge is that the timing of natural experiments does not necessarily align with university/government funding cycles. Researchers may become aware of an opportunity or be approached to conduct a natural experiment; however, baseline measures need to be complete before the intervention occurs and this often means short time frames that do not align with funding opportunities. Therefore, planning in advance to allow for long lead times before the commencement of refurbishment is advisable where possible. Funding bodies also need to be made aware of this possibility and allow some flexibility in the budget to accommodate events outside of the researchers’ control.

Furthermore, when the refurbishment proceeded, the park refurbishment budget was less than originally envisaged and therefore some aspects of the planned refurbishment that would have appealed to older children (e.g. large flying foxes, slides and climbing equipment) were not included in the final design. Changes in the nature of the intervention and associated timelines meant that the study evaluated an intervention substantially different to what was initially envisaged. Fortunately, the involvement of partners enabled the researchers to be more informed of the progress of the intervention and have a greater understanding of any foreseeable issues, thus enabling the study protocols to be adjusted accordingly.

Such changes in timelines may also impact on the suitability of the control site/condition. This could potentially be an issue if the control site was pre-selected based on having no planned changes to the site during the original study period, but the study period changes due to delays in the timing of the environmental intervention. This possibility needs to be anticipated in the conception and planning of any natural experiment. Importantly, there is a need for process evaluation of the intervention, to determine what was actually implemented [25]. This may involve comprehensive audits of the intervention and control sites pre- and post-intervention.

### Identifying suitable controls

The likelihood of identifying a control site that is perfectly matched to the intervention site is limited, potentially introducing biases related to outcomes [26]. Indeed, in the REVAMP study, although both the intervention and control parks were very large parks, the control park was a third of the size and was located in a higher SES area than the intervention park. This was unavoidable because no other park of a similar size and level of amenity to the intervention park was located in a low SES area. Nonetheless, the control park fulfilled several other selection criteria. It had similar baseline features to the intervention park (mainly open spaces and picnic areas), was accessible via a shared walking/cycling path, had a sealed walking/cycling path within the park and had no planned improvements or changes during the proposed study period. Previous natural experiment studies involving schoolyard playgrounds have observed changes being implemented to control sites during the study period resulting in them being unsuitable as a control site for follow-up measures [20].

It may be challenging to identify a perfectly matched control site with similar built environment characteristics to the intervention site that serves a similar population (based on population characteristics, such as socio-economic and demographic factors). The need to identify an appropriate control site that is geographically far enough apart to limit contamination is a further challenge. Therefore, a compromise may be necessary by identifying the most “suitable control” site.

### Data collection

Depending on the location or nature of the natural experiment and the data collection methods employed, issues such as weather conditions may have a significant impact on study outcomes (e.g. use of the site), data collection and study protocols, particularly in terms of scheduling.

For example, park visitation is highly dependent on weather (i.e. people mostly choose not to visit a park if it is raining). Therefore, to minimise the effects of weather on study outcomes, in the REVAMP study, measures were conducted in the same month at all three time-points and the study protocol stipulated that data collection should not occur during rainy conditions. Due to the refurbishment timeline, the REVAMP data collections occurred in mid-autumn, when weather in Melbourne can be unpredictable. The study team closely monitored weather forecasts in the days leading up to each data collection day. Due to wet weather, planned days of data collection were required to be rescheduled in advance and, on occasion, days were cancelled at 5 am (when the latest forecast for the day was published), just a couple of hours prior to data collection commencing.

Scheduling data collection around weather is a conundrum when the forecast is ambiguous or when a data collection day commences in fine conditions, but then unexpectedly becomes inclement. The questions faced by the research team in these circumstances were: Should the day’s data collection cease when the weather became inclement? Should the day’s data collection continue in order to preserve the study’s budget and minimise the difficulties involved with having to reschedule to another day? Or should data collection continue in the hope that the weather improves, but at the risk of potentially being unable to use the data due to inconsistencies with previous days and having to schedule an additional day anyway?

An additional complication in the REVAMP study was that localised weather patterns meant that conditions sometimes differed between the two park locations. This raised the question: If it is dry in one park but wet in the other, should these data be included or excluded? In the REVAMP study, if differing weather conditions in each park occurred, additional days were re-scheduled to try to capture more consistent weather at both parks.

Cancellation for any reason proved problematic as it was difficult to reschedule another day within the same month/season whilst avoiding planned events in the parks (e.g. school fun runs) and public holidays when outdoor family gatherings, barbeques and picnics in parks often occur. We avoided collecting data on these days as they may misrepresent typical park usage. Cancellation was particularly problematic on weekend days when there is less flexibility for replacement days. Some studies of the built environment may not be as dependent on weather or may be able to provide shelter (e.g. train stations). However, studies that are weather dependent need to plan for unforeseen weather by budgeting for additional data collection days and establishing detailed study protocols in the case of cancellation of data collection days.

### Staffing

The availability of reliable and trained staff is critical to the success of this type of research. The REVAMP study involved a large team of field staff (at least 12–15 per day) observing park users throughout the week from early morning through to late afternoon. Hence, ensuring an appropriate level of staffing was critical to data collection. There were a number of staffing considerations related to the quality of the data collection, the recruitment of study participants and practical logistical issues. This included: the provision of adequate training to ensure accurate and consistent data collection and measures to ensure compliance with organisational and ethical policies; efforts to make staff identifiable and trustworthy to enable recruitment of participants to the intercept interviews such as provision of uniforms, study materials and letters of authority; consideration of safety issues such as ensuring at least two staff were present in each target area at all times; and logistical issues such as cost-effective ways of transporting the research team to and from parks.

To ensure consistency, it is preferable that the same field staff collect data throughout the entire study. Having a sufficient pool of trained staff members to draw from to ensure adequate staffing on any given day, including back-up staff who can be called in at the last minute, whilst also guaranteeing each staff member enough shifts to warrant their involvement in the study, is necessary. Even with the best arrangements in place, unexpected disruptions to staffing such as illness, flat car batteries and even traffic accidents on the journey to the parks were all experienced in the REVAMP study. In addition, if a data collection day was cancelled due to poor weather and needed to be rescheduled, some staff were no longer available. This left the study short-staffed, necessitating rapid recruitment and training of new staff members to ensure data collection proceeded on suitable days. Natural experiment studies must anticipate a range of potential staffing issues and put contingency plans in place to ensure data collection is unaffected.

Budgetary constraints will likely dictate the overall staff numbers a study can afford, but given the importance of both reliable and available field staff, it is vital that this is given sufficient consideration. Importantly, there needs to be a contingency budget for staffing and allowance for additional days of data collection. Based on our experience, we would recommend 20% additional data collection days, over and above the projected number of days it would take to collect the data should there be no problems.

### Special events

Although more special events may occur in the intervention park after the refurbishment, to ensure consistency across control and intervention locations and between measurement time-points, it is important to avoid data collection on days which are atypical or inconsistent with other days such as when special or irregular events are being held. When considering natural experiments in the built environment these may include, for example, street parades, demonstrations, fun runs, road works, demonstrations, and markets.

In REVAMP, special events sometimes occurred in the park during the data collection period. These included a school fun run passing through a nominated target area and a “planned burn” as a part of the park’s fire management plan which caused significant smoke and would likely have deterred people from visiting the park. The two-way flow of knowledge from the partners in the REVAMP study provided the study team with greater awareness of planned events in the park than may have occurred without the inclusion of the partners. Maintaining regular communication between the study team and organisations responsible for the management of the intervention and control sites being studied is therefore vital, to enable the study team to plan for any special events and, where required, reschedule data collection well in advance to an alternative day. Monitoring the number of special events in the parks and how this changes over time may be additional measure of evaluation in future studies.

### Other considerations

An important consideration is to identify, record and if possible, eliminate any potential confounders that may impact the results of a natural experiment study. As described earlier, weather is an important confounder of park visitation, therefore baseline and follow-up observations should be conducted at the same time of the year to control for seasonal variation, and assessment of intervention and control groups should be conducted at the same time to enable accurate comparisons between groups. However, previous studies in parks that have been unable to conduct consistent measures across seasons have attempted to control for seasonal variation in the analyses [27].

In the REVAMP study, unsupervised equipment was placed at both parks to measure the use of walking/cycling paths. It was possible for park visitors to remove this equipment or move it to another location and park visitors could intentionally repeatedly pass by the counter to increase the number of counts. Managing unsupervised equipment is very difficult to control. Where possible, it is preferable that unmoveable equipment is installed and equipment must be regularly monitored to minimise errors or malfunctions and to re-install if moved.

In summary, it is important to attempt to anticipate and measure confounders that may impact the results observed that are independent from the intervention itself. This may be particularly challenging to record and monitor when the confounders are unanticipated.

## Conclusion

Natural experiments offer unique opportunities to evaluate the impact of large-scale changes to the built and natural environment. They provide valuable data that might not otherwise be possible to gather, because of the costs associated with modification of the environment. However, interventions evaluated through natural experiments are not implemented or controlled by researchers, and the settings in which they are undertaken are not always predictable. The conduct of REVAMP has provided insights that can be incorporated into the design, costing and management of future natural experiment studies. Factors beyond the control of the study team contribute to the complexity of both organising and conducting natural experiments, with potential flow-on effects to the quality of data. Therefore many extraneous factors need to be considered when designing, costing and conducting natural experiments; studies should identify opportunities to include key partners from the inception of the project, be flexible yet robust, and allow sufficient funding to accommodate unexpected changes in the research protocol.
